# Ovocalyxin-36 Is a Pattern Recognition Protein in Chicken Eggshell Membranes

**DOI:** 10.1371/journal.pone.0084112

**Published:** 2013-12-31

**Authors:** Cristianne M. M. Cordeiro, Hamed Esmaili, George Ansah, Maxwell T. Hincke

**Affiliations:** 1 Department of Cellular and Molecular Medicine, University of Ottawa, Ottawa, Ontario, Canada; 2 ISA North America, Division of Hendrix Genetics, Kitchener, Ontario, Canada; University of South Florida College of Medicine, United States of America

## Abstract

The avian eggshell membranes are essential elements in the fabrication of the calcified shell as a defense against bacterial penetration. Ovocalyxin-36 (OCX-36) is an abundant avian eggshell membrane protein, which shares protein sequence homology to bactericidal permeability-increasing protein (BPI), lipopolysaccharide-binding protein (LBP) and palate, lung and nasal epithelium clone (PLUNC) proteins. We have developed an efficient method to extract OCX-36 from chicken eggshell membranes for purification with cation and anion exchange chromatographies. Purified OCX-36 protein exhibited lipopolysaccharide (LPS) binding activity and bound lipopolysaccharide (LPS) from *Escherichia coli* O111:B4 in a dose-dependent manner. OCX-36 showed inhibitory activity against growth of *Staphylococcus aureus* ATCC 6538. OCX-36 single nucleotide polymorphisms (SNPs) were verified at cDNA 211 position and the corresponding proteins proline-71 (Pro-71) or serine-71 (Ser-71) were purified from eggs collected from genotyped hens. A significant difference between Pro-71 and Ser-71 OCX-36 for *S. aureus* lipoteichoic acid (LTA) binding activity was detected. The current study is a starting point to understand the innate immune role that OCX-36 may play in protection against bacterial invasion of both embryonated eggs (relevant to avian reproductive success) and unfertilized table eggs (relevant to food safety).

## Introduction

Multilevel, interactive defense strategies that function across biomineralized barriers are a hallmark of sophisticated biological structures. The calcareous avian egg, the hallmark of reproduction in birds, is a complex multilayered structure [1]. The eggshell resists physical and pathogen challenges from the external environment, while satisfying a variety of metabolic and nutritional needs of the developing embryo. Following ovulation, the forming egg traverses specialized regions of the oviduct where the egg white, eggshell membranes and eggshell are sequentially deposited in the magnum, white isthmus and uterine segments, respectively [2]. The innermost layer of the shell is the eggshell membranes that are deposited as a highly crossed-linked extracellular fibrous meshwork during ≤1.5 h passage through the white isthmus [1]–[3]. The fibres are organized into inner and outer membranes and are essential elements of a normal eggshell which will resist bacterial contamination [4], [5].

The eggshell membranes fibres are composed of highly cross-linked proteins such as collagens and cysteine-rich eggshell membrane protein (CREMP) [3], [6], [7]. These fibres serve as a structural support for enzymes and proteins that protect against invading microorganisms [1], [2]. Several studies have identified proteins in the eggshell membranes that possess antimicrobial activity, such as lysozyme and ovotransferrin [4], [8].

Ovocalyxin-36 (OCX-36) is a protein present in the uterine fluid collected during the active calcification stage of shell mineralization. It is present in the calcified shell, but particularly abundant in the eggshell membranes [9]. A polyclonal antibody against OCX-36 was used to expression-screen a hen uterine library, and a positive clone was sequenced and used for further hybridization screening. The resulting consensus sequence was subsequently assembled with ESTs to obtain a complete full-length cDNA [9]. The uterine OCX-36 message is strongly upregulated during eggshell calcification. OCX-36 expression occurs in the regions of the oviduct where eggshell formation takes place (isthmus and uterus), and also in the digestive tract [9], [10].

OCX-36 shares protein sequence homology, and similar exon and intron gene organization, with mammalian BPI and LBP proteins that have a major role in the innate immune response [9]. According to the new BPIFAn/BPIFBn systematic nomenclature for PLUNC proteins, the SPLUNC root has been replaced by BPIFA and the LPLUNC root was replaced by BPIFB. OCX-36 protein is a new member of BPIFB8 protein family [11].The OCX-36 gene is nested with in the BPI/LBP/PLUNC gene cluster on chromosome 20. However, the OCX-36 gene is highly specific to birds and is thought to have arisen by tandem duplication of an ancestral BPI/LBP/PLUNC gene cluster after the divergence of birds and mammals [10], [12]. LBP and BPI were the original members of the PLUNC protein family. These two protein members bind to LPS and play antagonistic functions in LPS mediated cellular signalling. Human LPB increases the inflammatory response induced by LPS whereas BPI shows antibacterial and anti-inflammatory functions [13], [14].In addition to its well known functions, BPI has anti-angiogenic activity, inhibits human endothelial cell growth and induces apoptosis [15], [16].The functional human PLUNCs are classified as short PLUNCs (SPLUNCs 1, 2 and 3) and long PLUNCs (LPLUNCs 1, 2, 3, 4 and 6) proteins. SPLUNCs have homology to the LPS- binding N-terminal domain of BPI, whereas LPLUNCs have overall homology to both the N-terminal and C-terminal domains of BPI. The N-terminal domain of BPI is responsible for its endotoxin neutralization and antibacterial activities while opsonic activity is associated with its C-terminal domain [17].

PLUNC and BPI proteins share similar functions. PLUNC proteins bind LPS, have bacteriostatic activity, induce bacteria agglutination and participate in cytokine production [18]. The common structural features that OCX-36 shares with BPI/LBP/PLUNC proteins are the basis for our hypothesis that OCX-36 participates in the innate immune response to pathogens as a pattern recognition protein [19].

Characterizing the biological function of OCX-36 protein will provide new insight into the natural defences of eggs which could mitigate the risk of food-borne disease for egg consumers. In this study, we have extracted, purified and characterized OCX-36 from eggshell membranes, as a first step to understand its functional role.

## Materials and Methods

### 1. Materials

*Staphylococcus aureus* ATCC 6538, *Listeria monocytogenes* ATCC 19112 and *Enterococcus faecalis* (clinical isolate), *P. aeruginosa* ATCC 15442, *Salmonella typhimurium* and *Escherichia coli* O111:B4 were obtained from Dr. Syed A. Sattar (Centre for Research on Environmental Microbiology, University of Ottawa). Luria-Bertani (LB) broth, bovine serum albumin (BSA) and casein were purchased from Bioshop Canada Inc. CM-Sepharose Fast Flow and DEAE-Sepharose Fast Flow resins were from Amersham Biosciences. Dialysis tubing (MWCO 12,000-14,000) was purchased from Fisher. Butyl, Pentyl, Hexyl, Octyl Agaroses and Concanavalin A Sepharose, phenol/chloroform/isoamylalcohol and Proteinase K were purchased from Sigma-Aldrich. Ni-NTA agarose was from Qiagen. Bio-Gel Hydroxyapatite (HTP), alkaline Phosphatase Substrate Kit and protein molecular weight marker (Blue Standards161-0373) were purchased from Bio-Rad. BCA protein assay reagent (bicinchoninic acid), 1-Step ABTS (1- 2′-azinobis-3-ethylbenzthiazoline-6-sulfonic acid) and protein molecular weight markers (SM0431) were purchased from Thermo Scientific. Anti-Rabbit IgG–horseradish peroxidase conjugate (HRP) conjugate was purchased from Promega. Western lightning Plus-ECl was purchased from Perkin Elmer. Biotinylated LPS and LPS from *E. coli* O111:B4, and LTA from *S. aureus* ATCC 6538 were purchased from InvivoGen. Streptavidin - alkaline phosphatase (SAP) was purchased from Invitrogen. Recombinant hLBP (rhLBP) was purchased from R&D Systems. The 96-well medium binding microplates were purchased from Costar, and 100-well honeycomb plates were purchased from Oy Growth curves. Amplitaq gold PCR master mix was purchased from Applied Biosystems.

### 2. Extraction of OCX-36

After extensive preliminary trials, an optimized protocol to extract OCX-36 from unfertilized chicken eggs was developed. “Standards eggs” used throughout method development and purification of OCX-36 were the same brand: BurnBrae Farms Super Bon-EE (oversized eggs that are 25% larger than regular large eggs), purchased from local groceries stores. The eggs (in batches of 5 to 10 dozen eggs) were broken and their contents were discarded. Eggshell interiors were rinsed under running demineralized water (DM). Eggshell membranes were manually stripped from the interior of the calcified eggshell and collected in DM. The wet membranes were sliced into smaller sizes for extensive rinsing with DM. Eggshell membranes were placed in 5 L of 1 M NaCl and stirred using an overhead mixer (IKA RW 20 digital, Cole-Parmer Canada) for 1 h at 4°C. The eggshell membranes were again rinsed using DM to remove any residual NaCl, followed by their collection on a Whatman No. 2 filter paper under vacuum suction. The moist membranes were weighed and added to the extraction buffer: 50 mM Tris-HCl, pH 8.5 containing 10 mM dithiothreitol (DTT) (30 mL of extraction buffer per gram of membrane). This extraction mixture was stirred (overhead mixer) to extract OCX-36 overnight at room temperature. The next day, the suspension was filtered (Whatman No. 2 filter paper) to remove large particles of membrane. This turbid solution containing extracted OCX-36 was centrifuged (3,500×g, 20 minutes, 4°C) to clarify and remove finer membrane particles.

### 3. Purification of OCX-36

The supernatant from the OCX-36 extraction was passed through two columns (CM-Sepharose Fast Flow and DEAE-Sepharose Fast Flow) connected in series. Both columns were pre-equilibrated with 50 mM Tris-HCl, pH 8.5 at a flow rate of 1 ml/min. After loading the supernatant onto the columns, the beads were washed with 50 ml of Tris-HCl, pH 8.5, 2 mM of DTT (all at 1 ml/min). After this washing, the CM- Sepharose column was disconnected and bound proteins were eluted from the DEAE column with 25 ml of 50 mM NaHepes, pH 7.0, 2 mM DTT, 350 mM NaCl at a flow rate of 1 ml/min. Eluted fractions (1 ml) containing OCX-36 were dialyzed and freeze dried.

### 4. Electrophoresis and Densitometry analysis of OCX-36

SDS-polyacrylamide gel electrophoresis (SDS-PAGE) was performed as previously [20], followed by staining with Coomassie Blue and destaining. Quantitative densitometric analysis of the stained bands was performed with Image Quant 300 and TL (GE Healthcare).

### 5. Western Blotting

OCX-36 samples were separated on 12% SDS–PAGE gels and electrotransferred to nitrocellulose membrane. The nitrocellulose membrane was washed in PBS-Tween 0.1% (10 mM sodium phosphate buffer, 0.154 M sodium chloride, pH 7.4, 0.1%Tween 20). The membrane was blocked with 3% BSA for 1 h, and then the membrane was washed in PBS-Tween 0.1% (2×5 min) and then incubated in PBS-Tween 0.1% for 1 h with primary antibody prepared against a 15-amino acid synthetic peptide corresponding to residues 51–65 of the mature OCX-36 protein, KHLQGMALPNIMSDR [9], diluted 1∶50,000 in PBS-Tween. After three washes in the same buffer, the membrane was incubated for 30 min with anti-rabbit IgG–HRP (1∶10,000), followed by washing (4 x PBS-Tween 0.1%, 2×0.1 M sodium phosphate buffer, pH 7.4). The membrane was then incubated with Western lightning Plus-ECl reagent. Immunoreactive bands were visualized with X-ray film for different exposure times.

### 6. Sample Preparation for Liquid Chromatography Tandem Mass Spectrometry (LC/MS/MS) analysis

Two different OCX-36 samples (preps 90 and 120) were resolved by 12% SDS-PAGE gels and lightly stained with Coomassie Blue following the recommended protocol. The gel pieces were sent to the Proteomics Platform of the Eastern Quebec Genomics Centre (Laval, QC) for LC/MS/MS analysis (include in-gel digestion, mass spectrometry analysis and Mascot data-base searching). The procedures for all these analyses were performed as previously described [21].

### 7. Data base searching and criteria for protein identification

MS/MS data were analyzed using Mascot (Matrix Science, London, UK; version 2.2.0), searching the uniref 100.2010.06.Gallus.gallus.9031 database, with trypsin digestion. Validation of MS/MS based peptide and protein identification were performed using Scaffold (version Scaffold- 3_00_08, Proteome Software Inc, Portland, OR). Protein identification was accepted at p = 0.05 probability, as specified by the Protein Prophet algorithm [22], [23], with at least 2 unique peptides (>95% confidence).

### 8. Bioinformatics analysis

The relative quantification of the identified proteins was calculated using the exponentially modified protein abundance index (emPAI) defined as 10^PAI^-1 [24].

### 9. Single Nucleotide polymorphisms (SNPs) in the OCX-36 gene

#### 9.1 Genomic DNA Purification

Chicken blood samples from pedigree White Leghorn laying hens (Hendrix Genetics) were assessed to identify birds possessing specific OCX-36 SNPs. Chicken blood samples were diluted in PBS, and then 50 to 100 uL of chicken blood cells were lysed by adding 500 uL of lysis buffer (10 mM Tris-HCl, 10 mM EDTA, 100 mM NaCl, 0.5% SDS and 20 µL/mL of mercaptoethanol) and vortexed for 30 min at 50°C. After 30 min of incubation, 5 µL of proteinase K (10 mg/mL) was added to the cell suspension and incubated overnight at 50°C. Genomic DNA was extracted by adding 600 µL of phenol/chloroform/isoamylalcohol, mixed by inversion 10 times followed by centrifugation at 14,300×g for 10 min at room temperature. After centrifugation, 500 uL of ethanol (100%) was added to the suspension containing DNA and centrifuged again at 14,300×g for 10 min at room temperature. DNA pellets were washed with 1 mL of cold ethanol and centrifuged again at 14,300×g for 10 min at 4°C. DNA pellets were dried overnight and then dissolved in 50 uL of sterile distilled water with incubation at 65°C for 30 min.

#### 9.2 SNP fine mapping analysis

SNP fine mapping analysis of genomic DNA prepared from pedigree White Leghorn laying hens was performed by Polymorphic DNA Technologies (Alameda, CA) using a “boost/nest” PCR. Boost and Nested primers were used for two step nested PCR (Table 1). The boost PCR reaction generated a larger fragment that was used as a template for the nested reaction. DNA (10 ng) was used for boost PCR reaction and 1 µL of boost product was a template for the nested PCR reaction. The PCR reactions were carried out using Amplitaq gold PCR master mix (Applied Biosystems). PCR conditions were: denaturation for 4 min at 94°C, annealing 25 min at 55°C, and extension 1 min at 72°C. DNA sequencing was done using the 3730/3730xl DNA analyzer machine (Applied Biosystems).

**Table 1:** Primer pairs used for boost/nested PCR.

Primers	Direction	cDNA region (5′ to 3′)	Size (bp)
Boost	Forward	ATCACCCCCTCTATTTG	302
	Reverse	GACGACCAACTGCATC	
Nested	Forward	CGTGGGTGCTGGAAA	347
	Reverse	CGGCAGCAGTGCTAT	

### 10. Antimicrobial Assays

#### 10.1 Viability assay

Overnight cultures were inoculated into LB broth and incubated at 37°C until an optical density of 0.2 at 600 nm was obtained. This bacterial suspension was centrifuged at 3000×g, 4°C for 10 min. The bacterial pellet was resuspended in PBS, to pH 7.4 to obtain 10^5^ CFU/mL. Bacteria were incubated with OCX-36 at 100 µg/mL in PBS for 1 h at 37°C. After incubation, the bacteria were serially diluted, plated on LB agar and then incubated for 24 h at 37°C to determine CFU's of surviving bacteria. Every dilution was performed in triplicate.

#### 10.2 Monitoring bacterial growth via the Bioscreen assay

This assay was performed in 100-well honeycomb plates containing 10^5^ CFU/mL and OCX-36 at 100 µg/mL in PBS, with incubation for 1 h at 37°C. After incubation, LB broth was added to each wells and the bacterial growth was monitored by optical density measurements at dual wavelength (420 nm–580 nm) every 15 min for 10 h using a Bioscreen C microplate reader. Results are shown as the average of at least three independent experiments.

### 11. LPS and LTA binding assays

#### 11.1 LPS binding assay using biotinylated LPS

The ability of OCX-36 to bind to lipopolysaccharide was measured using a modified plate-binding assay [25]. OCX-36 samples (100 uL) were incubated in 0.01% casein in PBS in a 96-well plate, overnight at 37°C. The wells were blocked with 300 uL of 0.1% casein in PBS and washed before adding the biotinylated *E. coli* O111:B4 LPS in 0.1% casein and PBS. Streptavidin- alkaline phosphatase in PBS was added for 30 minutes followed by washing and the addition of substrate. The rate of color development was monitored for 60 minutes by optical density measurements every 10 minutes at 405 nm–630 nm (dual wavelength mode). Recombinant hLBP was used as a positive control.

#### 11.2 ELISA-based LPS and LTA binding assays

The ability of OCX-36 to bind unmodified LPS and LTA was tested using a modification of a published protocol [26]. Briefly, 96 wells medium-binding microplates were coated with 100 µL of LPS from *E. coli* O111:B4 at 50 µg/mL or LTA from *S. aureus* ATCC 6538 at 0.5 µg/mL in PBS for overnight incubation at 4°C. The coated wells were blocked with 300 µL of PBS, 5% BSA (endotoxin :<2 EU/mg) for 1 h at 37°C. After washing the wells (three times) with PBS, 0.05% Tween-20 (PBS-T 0.05%), 100 µL of OCX-36 at 1.5; 5; 15 and 50 µg/mL in PBS were added to wells in triplicate and incubated at 37°C for 1 h. The wells were washed three times with PBS-T 0.05% and 100 µL of primary rabbit anti-OCX-36 antibody (1∶20,000 in PBS, 5% BSA) was added to wells and incubated at 37°C for 1 h. Following three washes, anti-rabbit IgG– HRP (1∶10,000 in PBS, 5% BSA) was added to wells and incubated at 37°C for 1 h. The wells were washed with PBS-T 0.05% before adding 150 µL of 2, 2′-azinobis-3-ethylbenzthiazoline-6-sulfonic acid (ABTS) substrate solution to each well. The rate of color development was monitored for 60 minutes by optical density measurements every 10 minutes at 405 nm–630 nm (dual wavelength, BIO-TEK model EL 311SL microplate reader), and is expressed as Abs/min.

### 12. Statistical Analysis

All experiments were carried out in triplicate and statistical significance was determined by Student's t-test with p<0.05 taken as significant. Results are reported as mean ± SEM.

## Results

### 1. OCX-36 extraction and purification

**Figure 1:**
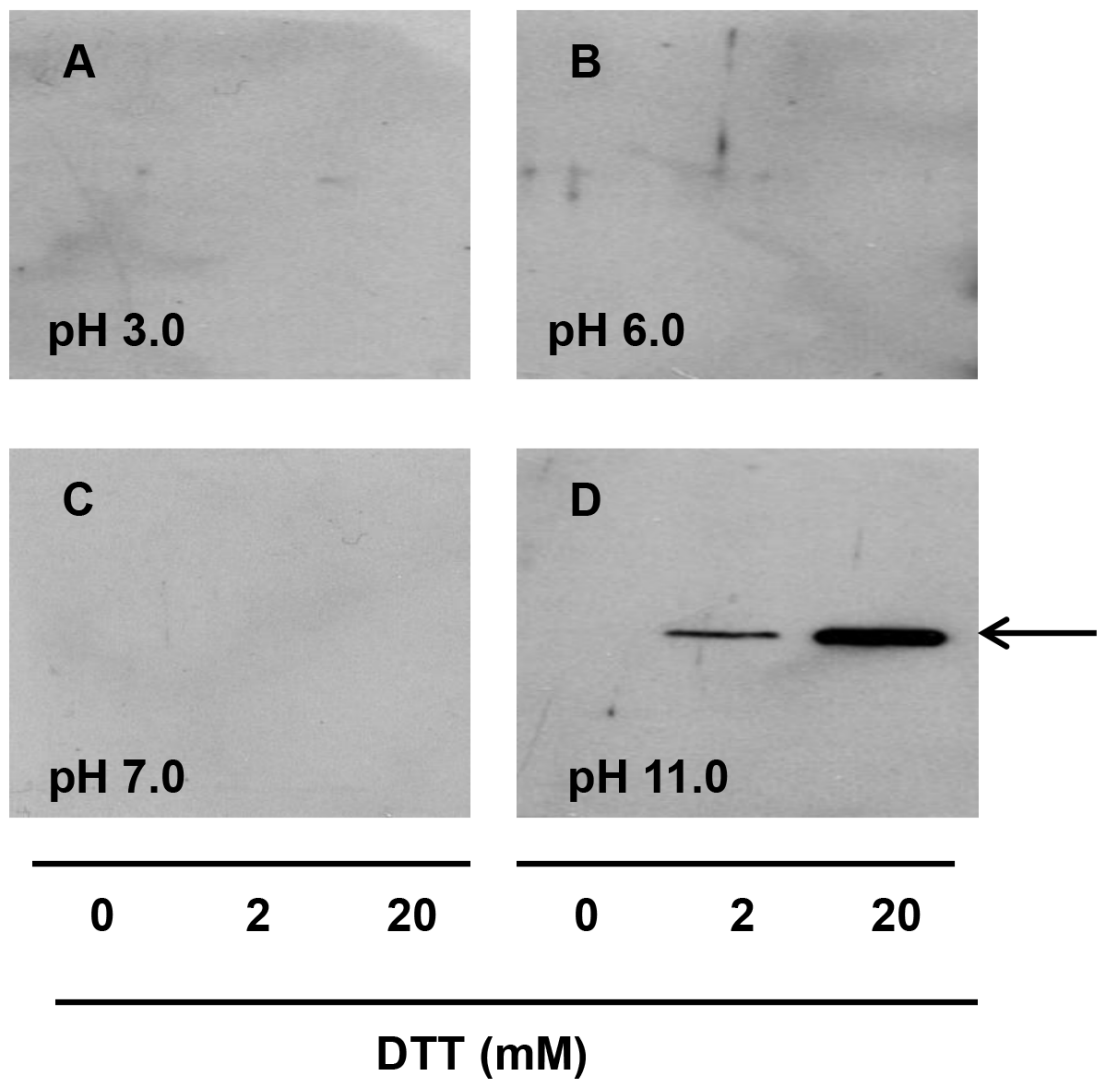
Western blot analysis for OCX-36 extracted using different extraction buffers. OCX-36 was extracted from eggshell membranes with different buffers at 0, 2 and 20 mM of DTT. (A) Buffer A (50 mM acetate, pH 3.0); (B) Buffer B (50 mM phosphate, pH 6.0); (C) Buffer C (50 mM phosphate, pH 7.0) and (D) Buffer D (50 mM Tris base, pH 11.0). Immunoreactive OCX-36 identified by Western Blotting (arrow).

**Figure 2:**
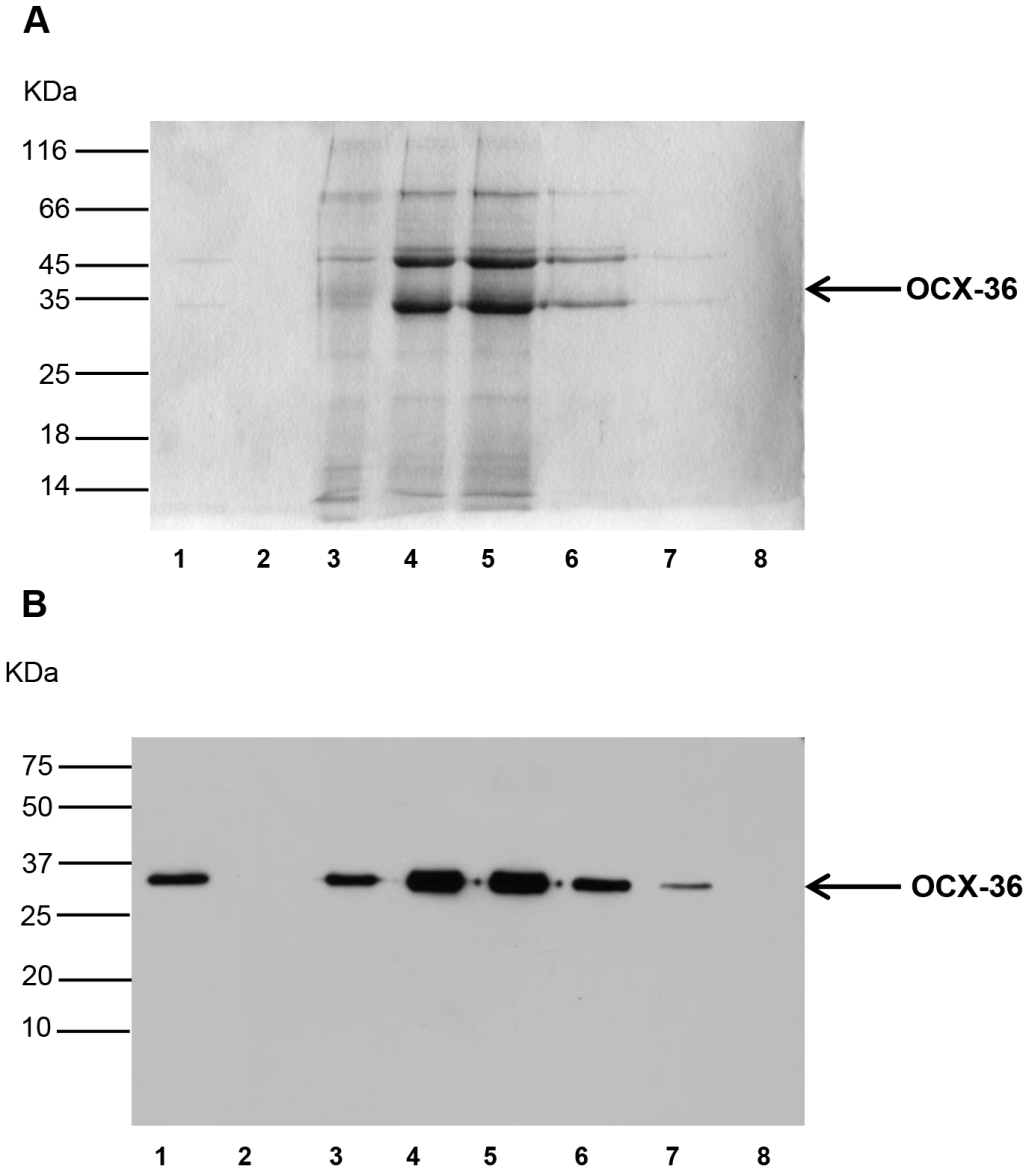
SDS-PAGE and Western blot analysis during OCX-36 purification. OCX-36 protein was extracted with buffer E (50 mM Tris-HCl, pH 8.5) and purified by CM-Sepharose Fast Flow and DEAE-Sepharose Fast Flow chromatographies. (A) 12.5% SDS-PAGE gel and (B) Western blot of OCX-36 fraction collected from each step of purification. The position of molecular weight standards (KDa) is indicated. Lane 1: Supernatant from overnight extraction prior to chromatography; Lane 2: Flow-through unbound to DEAE Sepharose; Lane 3: Sample prepared from DEAE beads before elution; Lanes 4–7: Fractions 1, 2, 3 and 4 eluted from the DEAE Sepharose with 50 mM Na Hepes, pH 7.0, 2 mM DTT, 350 mM NaCl; Lane 8: Sample prepared from DEAE beads after elution. Position of OCX-36 (Ovocalyxin-36) indicated by the arrow.

Different extraction buffers were tested to extract OCX-36 from eggshell membranes. The amount of extracted OCX-36 protein from eggshell membranes is dependent on the pH and DTT concentration used for the extraction buffer. Among all the tested buffers, buffer D and E yielded a detectable OCX-36 protein from eggshell membranes (Figure 1D and Figure 2B). Buffer E was selected as the buffer for further OCX-36 extraction to avoid harsh alkaline conditions. OCX-36 was characterized by SDS-PAGE and Western Blotting analysis which identified an approximately 33 KDa band (Figures 1 and 2).

**Figure 3:**
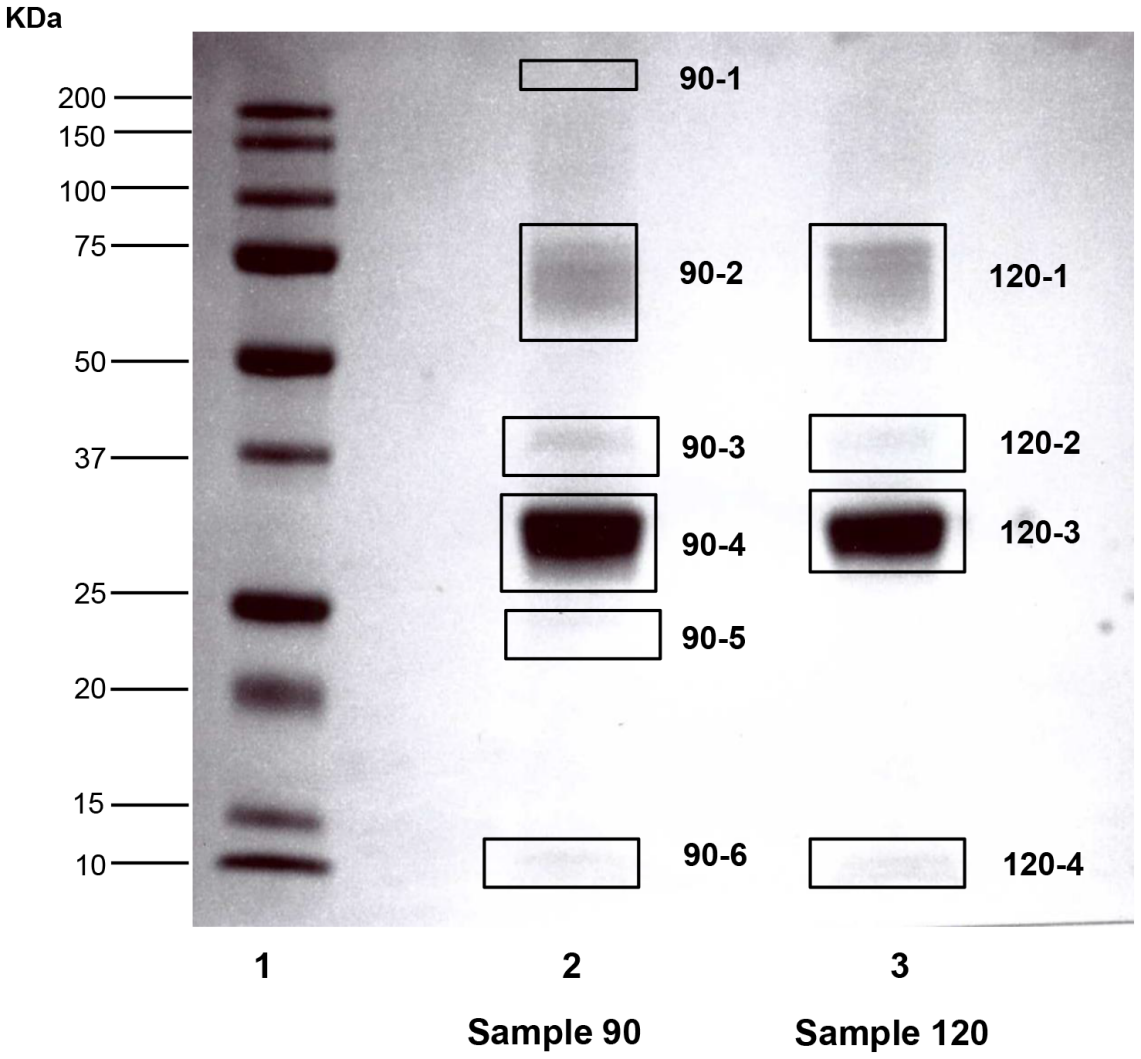
Proteomic analysis of purified OCX-36 samples (preparations 90 and 120). Boxes indicate the bands in the 12.5% Coomassie Blue - stained gel that were cut out. The excised bands were sent for LC/MS/MS sequencing analysis (methods). Samples 90 and 120 are two individual preparations of OCX-36. Lane 1: Molecular weight standards (KDa); Lane 2: Sample 90; Lane 3: Sample120.

**Figure 4:**
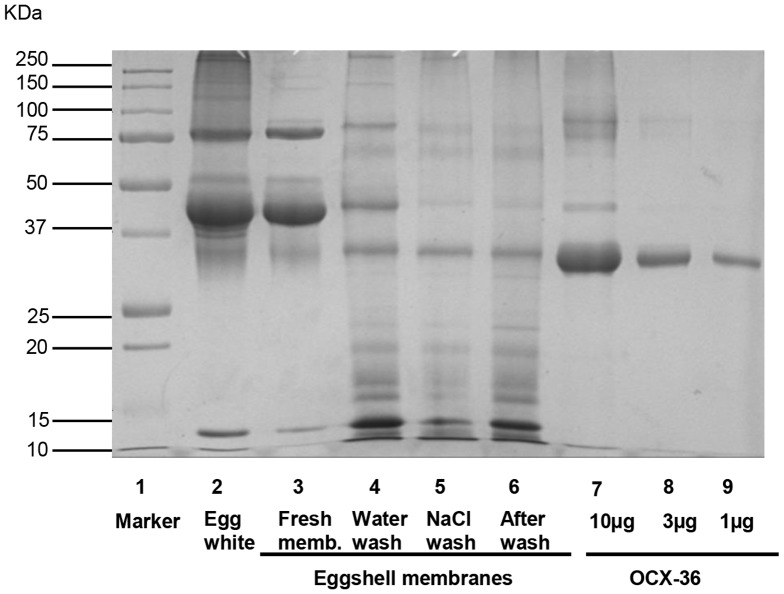
OCX-36 enrichment during extraction and purification. Samples from each stage of OCX-36 purification were prepared for SDS-PAGE by heating in Laemmli buffer, followed by determination of protein concentration for the extracted proteins (from membranes). All samples are 10 ug, except as indicated. Lane 1: Molecular weight standards (KDa) are indicated. Lane 2: Egg white; Lane 3: Fresh – sample from eggshell membranes prior to stripping from eggshell interior; Lane 4: Water wash - sample from eggshell membranes after extensive washing with water; Lane 5: NaCl wash – sample from eggshell membranes after washing with NaCl; Lane 6: After extract – membranes after OCX-36 extraction with buffer E, revealing unextracted proteins; Lane 7–9: Purified OCX-36 (after sequential CM and DEAE chromatography). The purity of OCX-36 at the final purification step (1 µg) was estimated as 98%.

The purification of extracted OCX-36 was carried out using ion exchange chromatography after investigating a variety of purification methods which were unsuccessful since OCX-36 was not retained by the resin (Table 2). Successful purification of OCX-36 protein was accomplished using a two-step procedure. The first step was to remove fine membrane particles from the OCX-36 extraction supernatant and also retain positively charged egg white proteins (i.e. lysozyme), by using the CM-Sepharose column as a guard column. OCX-36 protein passed through the CM-Sepharose column and was retained by the DEAE-Sepharose resin, from which the relatively pure protein could be eluted with a step-gradient of 0.25 M NaCl. At this stage, eluted fractions from the DEAE-Sepharose column resulted in an OCX-36 immunoreactive band with an apparent molecular weight around 33 kDa (Figure 2A and 2B). LC/MS/MS analysis and Mascot database for protein identification confirmed the immunoreactive band as OCX-36 (Figure 3, Table 3). The comparison between OCX-36 contents during extraction and purification steps was performed by densitometry using Image Quant software. Most of the extractable proteins from the fresh eggshell membrane starting material were egg white proteins that include ovotransferrin, ovoalbumin and lysozyme whereas; these were almost completely removed from the purified OCX-36. Calculations indicated that OCX-36 in the purified material was approximately 98%, and that the degree of purification was about 1000-fold (Figure 4, Table 4).

**Table 2:** Purification methods investigated for OCX-36 purification.

Chromatography technique	Types
Hydrophobic interaction chromatography	Butyl, Pentyl, Hexyl and Octyl (Agarose)
Carbohydrate binding	Concanavalin A
Affinity Chromatography	Immobilized Nickel
Hydroxyapatite	

**Table 3:** Merged proteomic results for purified OCX-36 samples using two independent samples.

Identified	Accession	MW	No. unique	emPAI^b^	No. unique	emPAI^b^
Proteins	Number	(kDa)	peptides^a^		peptides^a^	
			prep 90		prep 120	
Ovocalyxin-36	Q53HW8	49	7	3.01	7	3.37
Ovalbumin	P01012	43	11	3.21	5	0.49
Ovalbumin-related protein Y	UPI0000E7FE38	44	4	0.37	-	-
Tiarin-like	Q25C35	56	7	0.54	3	0.2
Actin, cytoplasmic type 5	P53478	42	4	0.39	-	-
Hypothetical protein	UPI0000E806B4	76	-		2	0.1
BPI-like 2	UPI0000E7F8E6	44	2	0.17	-	-
Ig mu chain C region	P01875	49	2	0.15	-	-
IG heavy chain variable region	UPI0000ECBF72	13	2	0.67	-	-
Tenascin	F1N8F4	204	2	0.03	-	-

Proteomic analysis was performed on the bands identified in Figure 4, followed by merging the peptides (Mudpit analysis) to assess the total protein constituents and estimate their relative abundance (emPAI).

^a^ Pro-71 (GLLSSPTIITGLHLER) and Ser-71 (GLLSSSTIITGLHLER) were combined.

^b^ Exponentially modified protein abundance index.

**Table 4:** Densitometry analysis of OCX-36.

Extraction and Purification steps	Estimated OCX-36 content (%)	Fold Purification
A. Stripped membranes	<0.1%	1
B. Membranes: water wash	9%	90
C. Membranes: NaCl wash	15.6%	156
D. Purified OCX-36	98%	980

The average weight of eggshell membranes stripped from 60 eggs was 27.5±4.9 g (0.44 g/egg) and the yield of purified OCX-36 protein was 0.33±0.08 mg OCX-36/gram of eggshell membranes (0.13 mg/egg). The total amount of OCX-36 samples extracted and purified from standards eggs over all experiments (approximately 90 preparations) was 843 mg.

### 2. Polymorphisms in OCX-36 gene

Data base analysis of the NCBI chicken genome (www.ncbi.nlm.nih.gov/snp) identified rs15177583 predicting a non-synonymous SNP (conferring a C-T substitution leading to a change at amino acid position 71 (Proline-71/Serine-71). This polymorphism was apparent in the proteomic sequencing data obtained with OCX-36, indicating that our typical purification from 5 – 10 dozen standard eggs had purified a mixture of the ser/pro versions (Table 3). This suggested that individual birds could be identified which laid eggs containing either the Pro-71 or Ser-71 polymorphic forms. SNP fine mapping of genomic DNA isolated from individual White Leghorn pedigree birds confirmed the predicted mixture of genotypes (Table 5). Two distinct populations of birds were examined. Their genetic background differed in that one was a fast feathering population and the other was a slow feathering population which had undergone many generations of selection for egg production and related traits. We observed the presence of homozygous C alleles coding for proline, homozygous T alleles coding for serine and heterozygous Y coding for either proline or serine. The homozygous C alleles coding for proline-71 was most represented in these animals (61.3%)(Table 5). However, significant differences in the abundance of the alleles were observed between the two (slow/fast feathering) populations. This trait is likely unrelated to OCX-36 polymorphism, but rather reflects a founder effect. Birds homozygous for each allele were identified and caged individually for egg collection. OCX-36 was extracted and purified from eggshell membranes harvested from eggs laid by birds tested that were homozygous for the OCX-36 alleles coding for either Pro-71 or Ser-71.

**Table 5:** Allelic frequencies of OCX-36 SNPs in Pedigree White Leghorn birds.

Bird i.d.	Alleles (polymorphism)	Number of birds	%
1–40^a^	Heterozygous Y (OCX-36)	18	45
	Homozygous C (Pro-71)	10	25
	Homozygous T (Ser-71)	12	30
41–80^b^	Heterozygous Y (OCX-36)	1	2.5
	Homozygous C (Pro-71)	39	97.5
	Homozygous T (Ser-71)	0	0

^a^ Slow feathering group

^b^ Fast feathering group

### 3. Antimicrobial activity of purified OCX-36 from standard eggs

**Figure 5:**
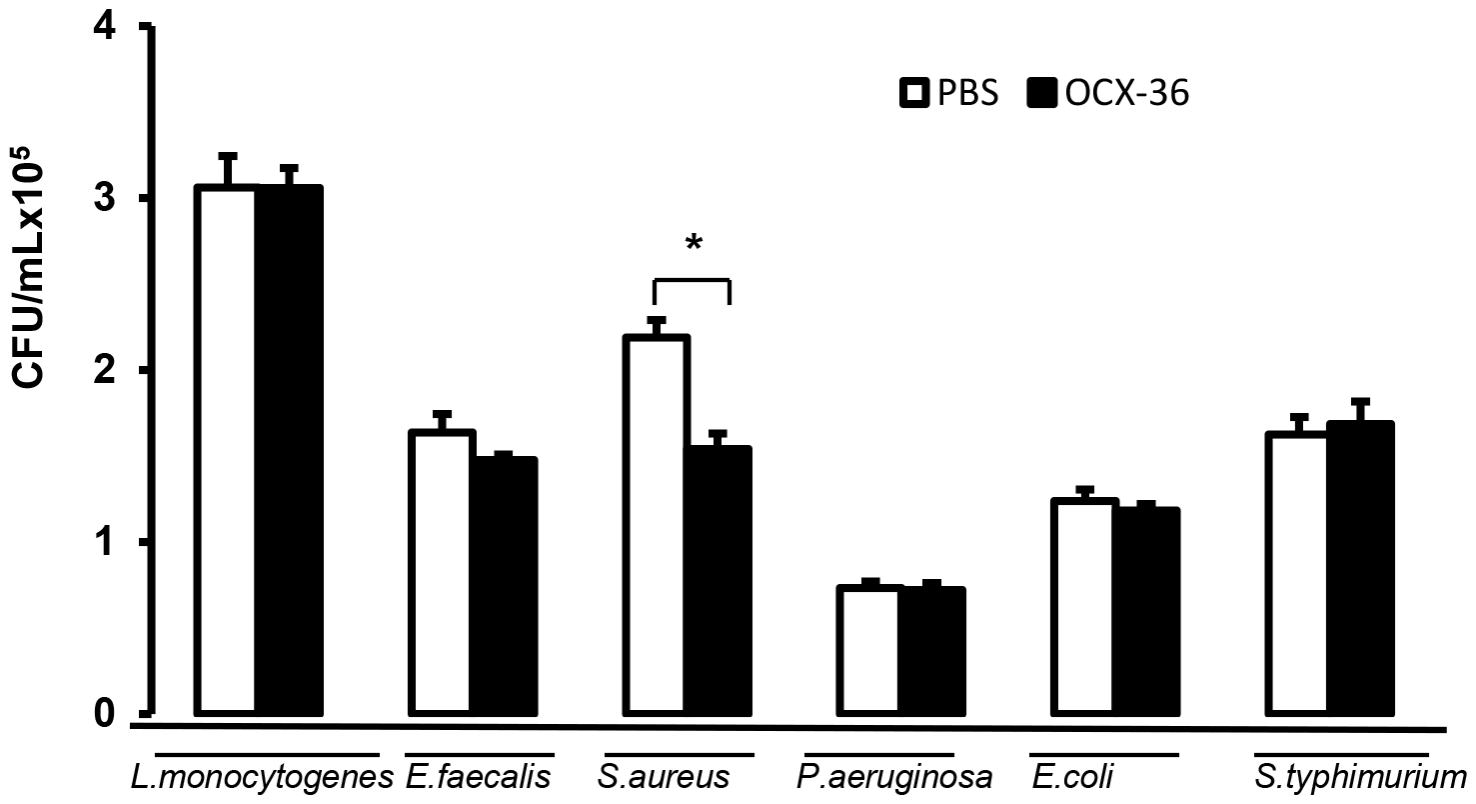
Antimicrobial activity of OCX-36 against Gram-positive (*L. monocytogenes, E. faecalis* and *S. aureus*) and Gram-negative bacterial strains (*P. aeruginosa, E.coli and S. typhimurium*). OCX-36 only showed antimicrobial activity against *S. aureus* ATCC 6538 which was significantly different from the vehicle control (PBS) (* p<0.001). The results are the average of three independent experiments, each performed in triplicate.

**Figure 6:**
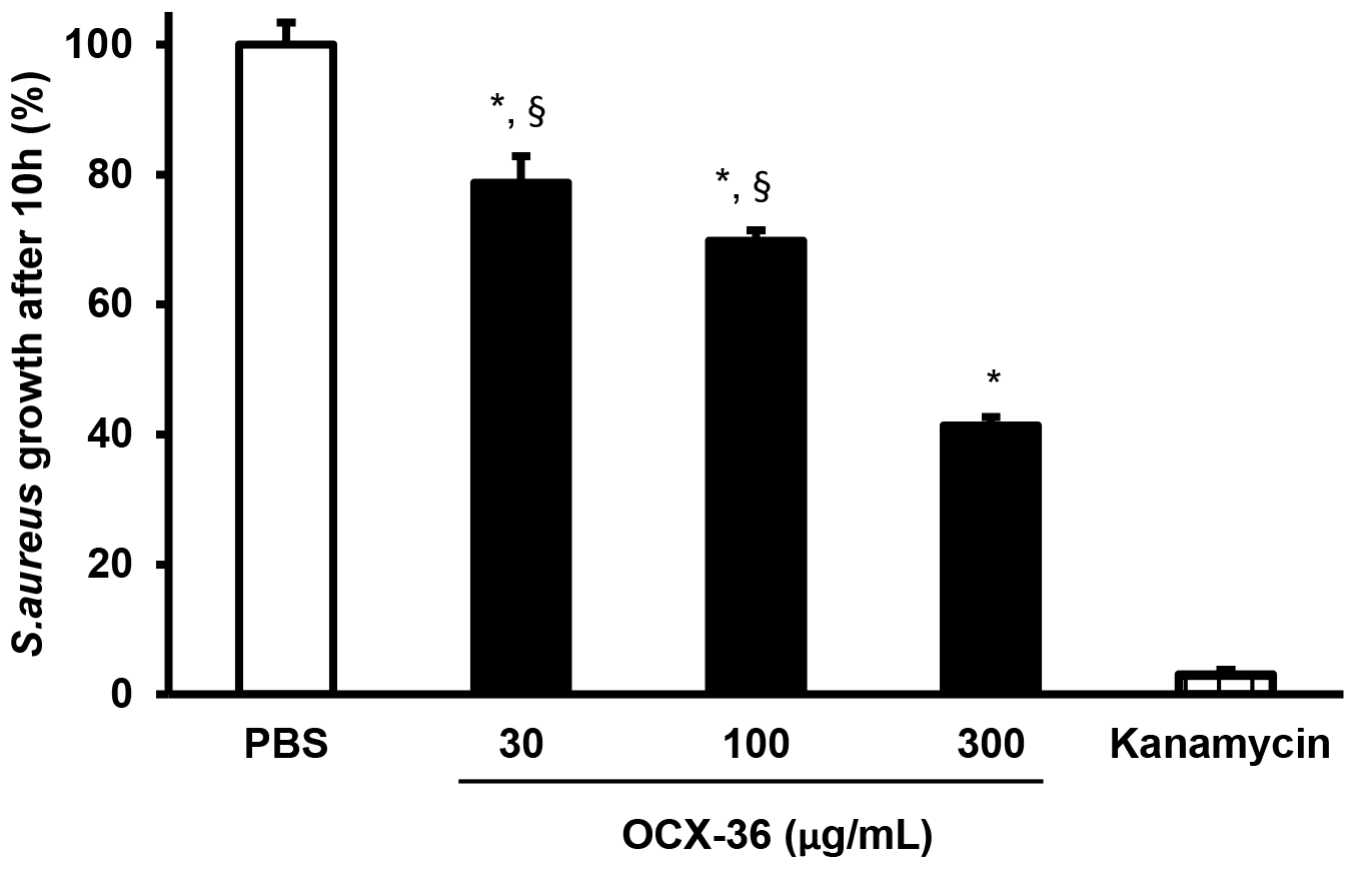
Bacteriostatic activity of OCX-36 against *S.aureus* ATCC 6538. Bacterial growth was analyzed using the Bioscreen C microplate reader over 10-36 at 30, 100 and 300 µg/mL was found to be significantly different from the vehicle control (PBS) (* p<0.05). OCX-36 at 30 and 100 µg/mL was statistically different from OCX-36 at 300 µg/mL (§ p<0.05). The results are the average of three individual experiments, each performed in triplicate.

Purified OCX-36 protein at 100 µg/mL was able to cause a significant (* p<0.001) inhibition of *S. aureus* ATCC 6538 cell viability providing bactericidal effect during 24 h of exposure compared to vehicle control (*S. aureus* 10^5^ CFU/mL, PBS) (Figure 5). OCX-36 at 30 µg/mL as well as 100 and 300 µg/mL (* p<0.05) showed a significantly bacteriostatic activity against *S.aureus* for 10 h of incubation compared to vehicle control. OCX-36 at 300 ug/mL was significantly different from OCX-36 at 30 ug/mL and at 100 ug/mL (§ p<0.05) (Figure 6).

### 4. Antimicrobial activity of OCX-36 from genotyped hens

**Figure 7:**
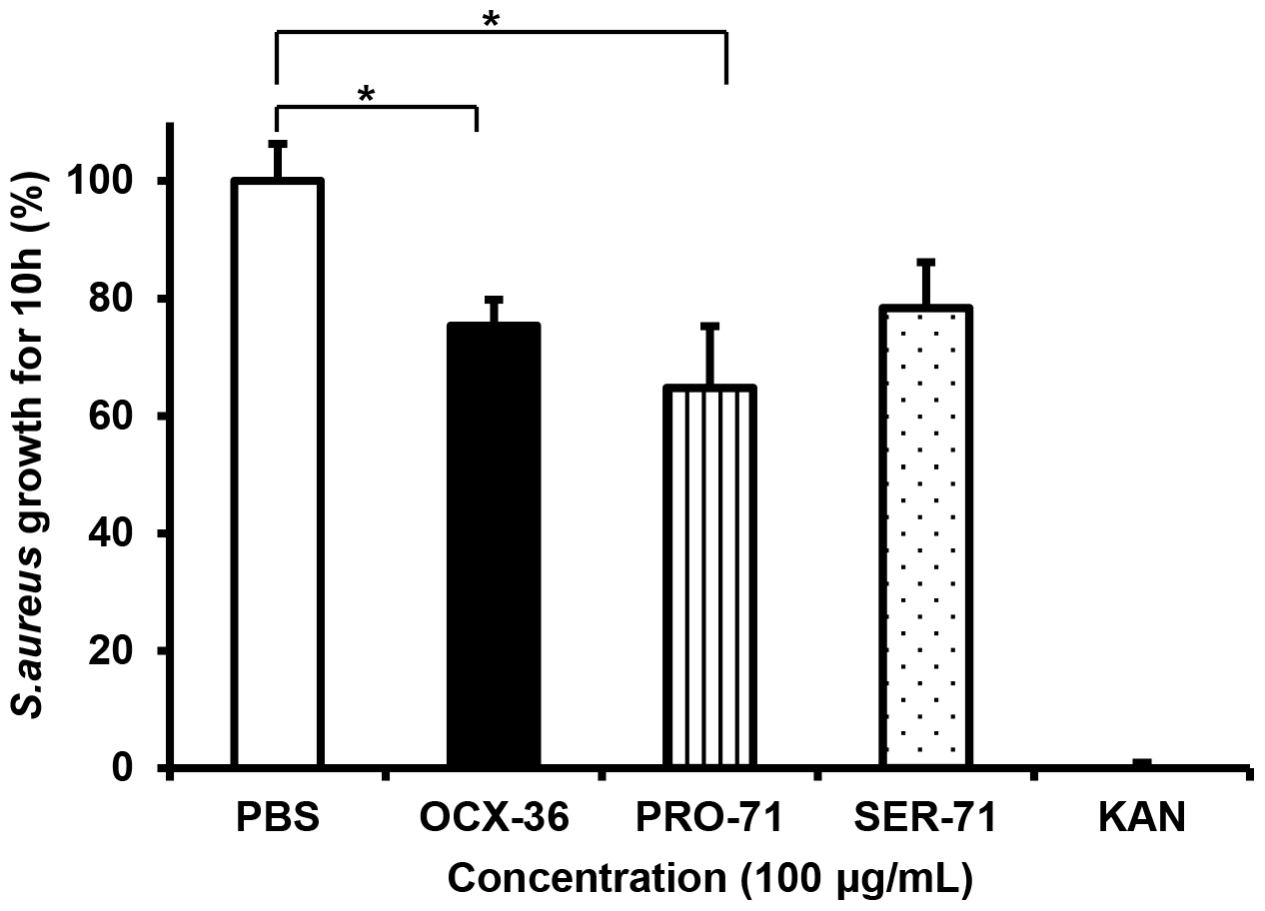
Antimicrobial activity of OCX-36 polymorphic forms from genotyped eggs (Pro-71 and Ser-71), and the OCX-36 mixture from standard eggs against *S. aureus* ATCC 6538. *S. aureus* growth was inhibited by OCX-36 (mixture of Pro-71 and Ser-71) and the individual forms at 100 µg/mL for 10 h of incubation, as assessed with the Bioscreen C microplate reader (compared to vehicle control growth in PBS) (* p<0.05).The results are averages of three individual experiments, with each experiment performed in triplicate.

Both versions of OCX-36 (Pro-71 and Ser-71) at 100 µg/mL showed significant bacteriostatic activity against *S. aureus* ATCC 6538 compared to vehicle control (10^5^ *S. aureus* CFU/mL, PBS). Both polymorphic forms of OCX-36 were also significantly bacteriostatic against *S. aureus* for 10 h of incubation (*p<0.05) (Figure 7).

### 5. LPS – binding activity of purified OCX-36

**Figure 8:**
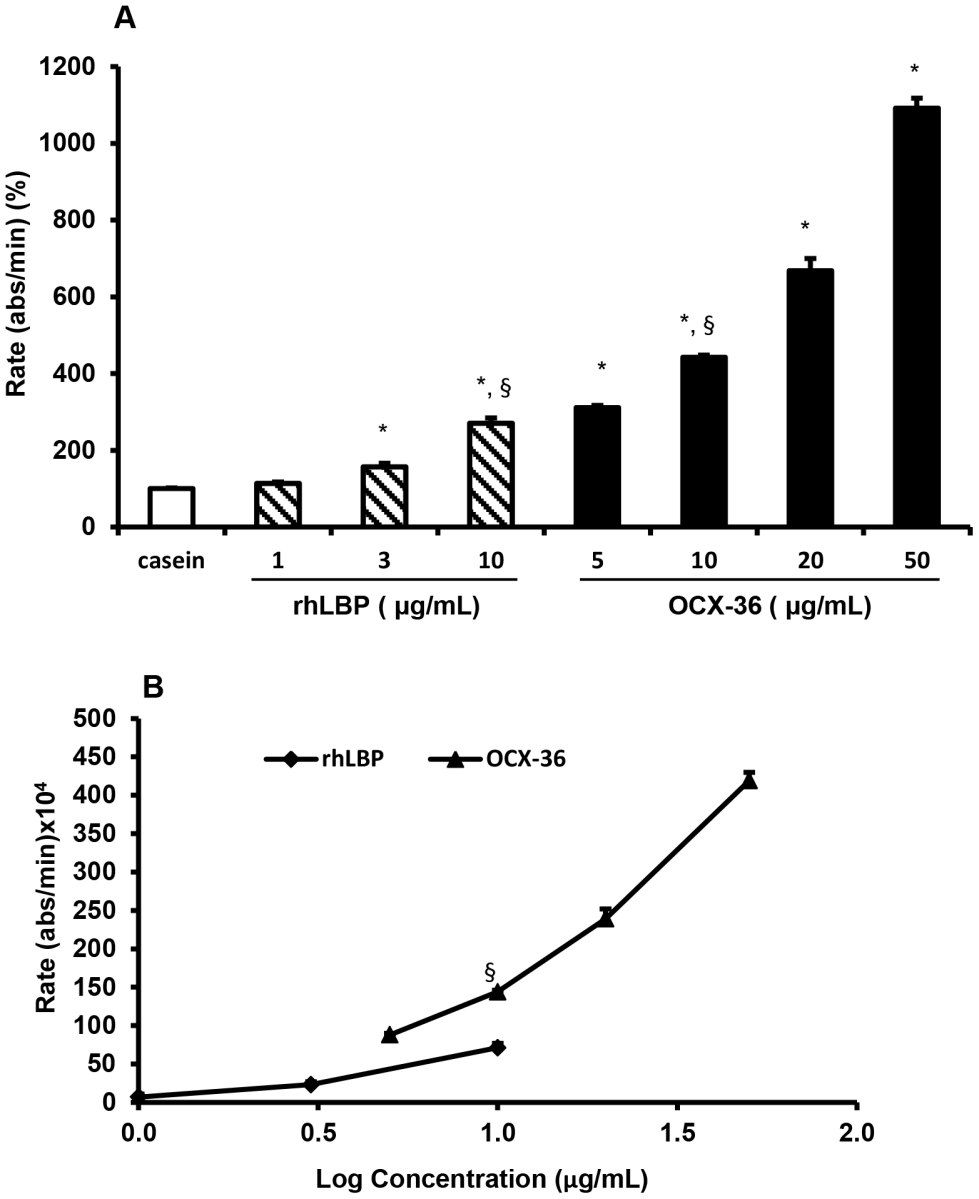
LPS binding activity of purified OCX-36 and rhLBP towards biotinylated *E.coli* O111:B4 LPS. The LPS binding activity of OCX-36 purified from standard eggs (5, 10, 20 and 50 µg/mL) and recombinant human Lipopolysaccharide binding protein (rhLBP) (1, 3 and 10 µg/mL) was tested using the biotinylated LPS plate-binding assay. (A) The LPS binding activity of rhLBP and OCX-36 were significantly different than the negative control, casein (* p<0.05). (B) OCX-36 and rhLBP proteins showed a significant binding towards biotinylated *E. coli* O111:B4 LPS and OCX-36 was significantly higher than the positive control, rhLBP at 10 µg/mL (§ p<0.05). The LPS binding activity of both protein were normalized to casein. The results are three individual experiments and each experiment was performed in triplicate.

**Figure 9:**
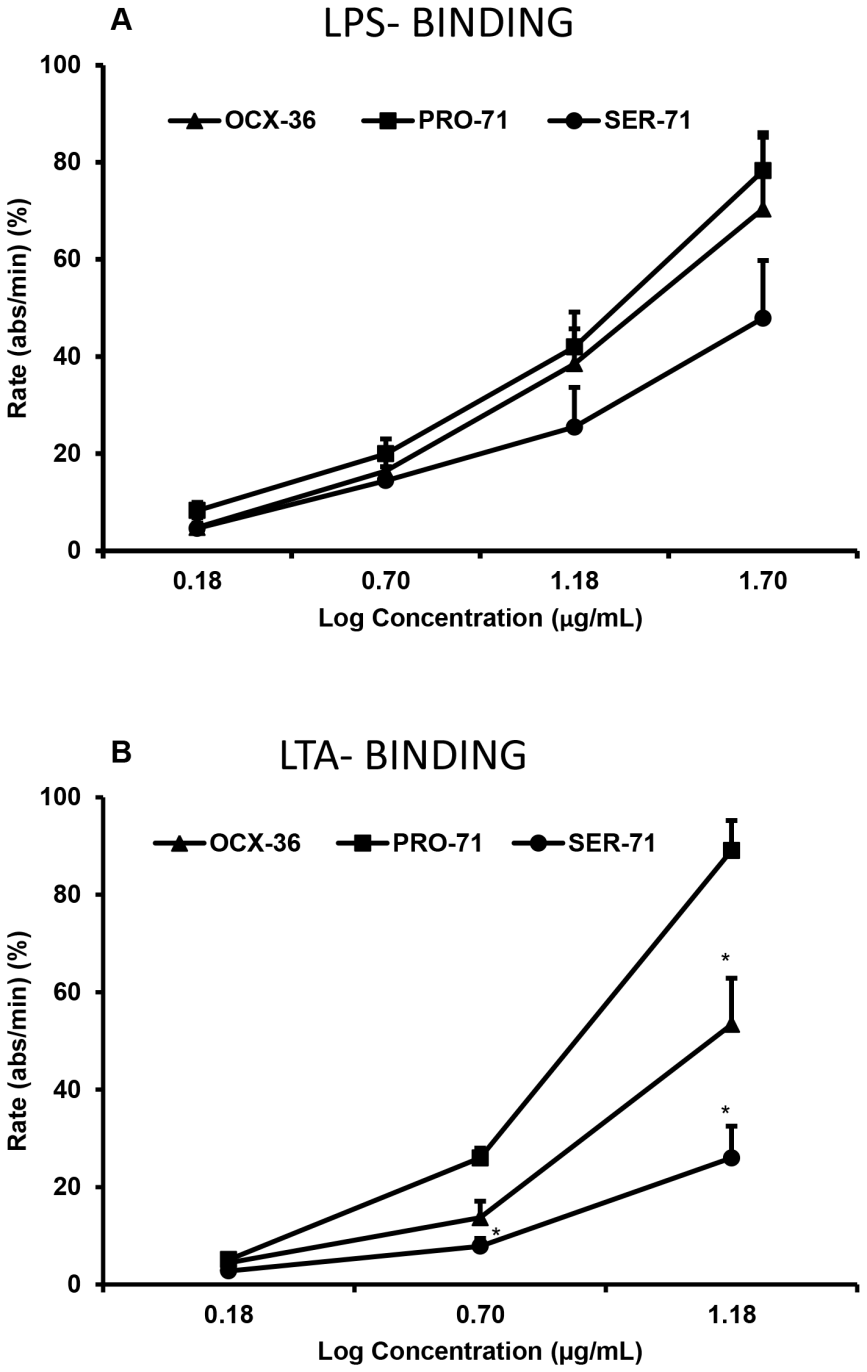
LPS and LTA binding activity of OCX-36 (mixture) and OCX-36 individual forms (Pro-71 and Ser-71). The LPS and LTA binding to OCX-36 (mixture), OCX-36 (Pro-71) and OCX-36 (Ser-71) were tested with the ELISA binding assay. Binding activity was corrected for background BSA binding and individual experiments were normalized before merging. (A)LPS-BINDING: All proteins tested showed *E. coli* LPS binding activity at 1.5, 5, 15 and 50 µg/mL but a significant difference was not observed between them at any concentration. All concentrations of Pro-71 showed significant different LPS binding activity between them (p<0.05). (B) LTA-BINDING: The LTA binding activity of OCX-36, Pro-71 and Ser-71 were tested at 1.5, 5 and 15 µg/mL. The LTA binding activity of OCX-36 (15 µg/mL) and Ser-71 (5 and 15 µg/mL) were significantly different than the Pro-71 (*p<0.05). All concentrations of Pro-71 showed LTA binding activity significant difference between them (p<0.05). The LTA binding activity of OCX-36 at 5 and 15 ug/mL were significant different between these concentrations (p<0.05). The results are the average of three individual experiments, with each experiment performed in triplicate.

The ability of OCX-36 to bind LPS was investigated. OCX-36 protein at 100 µg/mL showed significant binding towards biotinylated *E. coli* O111:B4 LPS in a dose dependent manner, which was significantly higher than the positive control, rhLBP (Figure 8). Control tests of LPS binding to purified ovalbumin and ovotransferrin at 100 µg/mL were also tested (data not shown). Ovotransferrin exhibited LPS binding activity but this protein was not detected in purified OCX-36 contents by MS/MS analysis. Although ovalbumin was detected in purified OCX-36 preparations by MS/MS analysis, controls using purified ovalbumin found that it did not bind to biotinylated *E. coli* LPS O111:B4. The LPS binding activity of OCX-36 from standard eggs was also confirmed using non-biotinylated LPS binding activity assay (Figure 9A).

### 6. LPS and LTA activity of OCX-36 from standard and genotyped eggs

Purified OCX-36 (mixture of Pro-71 and Ser-71 forms) and individual OCX-36 polymorphic forms showed significant *E. coli* O111:B4 LPS binding, but there was no significant difference in binding between these proteins (Figure 9A). We also tested *S. aureus* LTA binding activity (Figure 9B). The Pro-71 form of OCX-36 showed significantly higher LTA binding activity than both mixed OCX-36 and Ser-71 protein at 15 ug/mL. Mixed OCX-36, containing both the Pro-71 and Ser-71 forms, showed an intermediate LTA binding activity (Figure 9B).

## Discussion

### 1. OCX-36 identification and purification

OCX-36 is an abundant protein in eggshell membranes, where it is readily detected by immunofluorescence techniques, leading to speculation about its role in defence of the developing embryo and in unfertilized table eggs against pathogenic bacteria [9]. This previous work underscores the relevance of the current study to investigate the biological function of OCX-36 in chicken eggs. A key feature of the current study was to develop a suitable method for extraction and purification of OCX-36 protein from eggshell membranes. DTT was found to be an essential component of the extraction buffer. In previous studies on chicken egg proteins, reducing agents such as 2-mercaptoethanol and DTT, have been frequently used to break intra- and inter- molecular disulfide bonds [27], [28]. The efficient extraction of OCX-36 by DTT leads us to speculate that association of OCX-36 protein with chicken eggshell membranes is stabilized by disulfide bonds. After separation of extracted and purified proteins by SDS-PAGE, an immunoreactive OCX-36 band at approximately 33 kDa (confirmed by proteomic analysis) was detected (Figure 2A), which is slightly lower than the originally identified band at 36 kDa [9]. Differences in the molecular weight markers and electrophoretic conditions (i.e. % acrylamide) between these studies are likely at the origin of these differences in apparent molecular weight. Indeed, in our previous study we proposed a hypothesis for the differences between sequence molecular weight of OCX-36 and that detected by SDS-PAGE. For example, possible oligomerization of OCX-36, cross-linking (intramolecular) or even abnormal mobility on SDS-PAGE due to its highly hydrophobic amino acid sequence [9].

The next step for OCX-36 characterization was purification using ion exchange chromatography. This chromatography method has been extensively used to purify egg proteins because it may not affect the protein structure and the purified proteins remain active [29], [30]. The optimized purification method used for OCX-36 was able to retain and elute OCX-36 from the DEAE-Sepharose column with >98% purity (Figure 2A and 2B) as demonstrated by densitometric analysis (Table 4). Further LC/MS/MS analysis was performed and identified OCX-36 as the most abundant protein. Although, this extraction and purification scheme yielded essentially pure OCX-36 (>98%), other proteins were detected when large amounts of protein were analysed by SDS-PAGE. Most protein contaminants identified in purified OCX-36 samples by MS/MS analysis were egg white proteins (Table 3), of which ovalbumin was the most abundant. These egg white proteins were also found in other compartments such as oviduct fluid and calcified eggshell matrix. However, egg proteins such as Actin cytoplasmic type 5, Tiarin-like and BPI-like 2 were also identified. Certain of these proteins are thought to be nonspecific contaminants that are derived from other oviduct segments or luminal cells during egg formation [31], [32]. Lysozyme, a well-known and potent antimicrobial protein, was not detected in preparations of purified OCX-36.

### 2. Antimicrobial activity and LPS/LTA binding activity of OCX-36

The natural resistance of the contents of the avian egg to contamination by pathogens depends upon the physical barrier of the eggshell and upon chemical defences due to antimicrobial egg proteins and peptides that are secreted by the luminal cells of the oviduct and become incorporated into egg compartments such as egg white, eggshell membranes and the eggshell [33].

The recognition that avian OCX-36 is a member of the BPI/LBP/PLUNC gene locus, and that its protein sequence has similarity to mammalian BPI/LBP, was the basis for our hypothesis that OCX-36 has an antimicrobial activity [9], [19]. BPI supresses LPS inflammatory activity by binding its N-terminal domain to lipid A moiety in LPS [34]. This interaction of N-terminal domain of BPI to LPS is crucial for its antimicrobial and anti-inflammatory activities of BPI [35], [36]. BPI binds to bacterial LPS and penetrates the inner membrane of bacteria to causes depolarization of its membrane and cell death [37].

We evaluated the antimicrobial activity of purified OCX-36 against a variety of Gram-positive and Gram–negative bacteria, and assessed its capacity to bind LTA and LPS. The results of viability and growth inhibition assays showed that OCX-36 was only effective against *S.aureus* ATCC 6538. OCX-36 possesses both bactericidal and bacteriostatic effects against *S. aureus* ATCC 6538. The bacteriostatic effect of OCX-36 against *S.aureus* ATCC 6538 was dose and time dependent.

As predicted from their structural homology to BPI, PLUNCs have been demonstrated to possess antimicrobial activity and anti-inflammatory properties [38], [39]. Certain PLUNC proteins showed antimicrobial activity against specific airways pathogens such as *P. aeruginosa* and *Mycoplasma pneumonia* [40]. Moreover, SPLUNC 1 was able to inhibit *P. aeruginosa* growth and binds to LPS [41]. A proposed model for the antimicrobial action of SPLUNC 1 against *P. aeruginosa* in vitro is that SPLUNC 1 protein increases bacterial cell permeability and has a chemoattractant effect upon macrophages and neutrophils at the site of infection [42]. SPLUNC 1 showed a significant antimicrobial activity against *Mycoplasma pneumonia* but only modest inhibition of *E. coli* growth [43].

Recombinant human BPI (rhBPI) is able to directly interact with and neutralize LPS derived from different Gram negative bacteria [44], [45]. The N- terminal of rhBPI at low concentrations in biologic fluids such as serum and whole blood showed antimicrobial activity against Gram-negative bacteria including *E. coli*, *Salmonella typhimurium*, *Shigella* and *Enterobacter* spp. [45]. The rhBPI 21-KDa protein has been used for the treatment of children with meningococcal sepsis [46]. Recombinant BPIs from human and mouse have the ability to neutralize LPS from Gram-negative bacteria. However, mouse recombinant BPI does not inhibit *P. aeruginosa* growth even at higher concentrations than human recombinant BPI [47].

BPI proteins and peptides from BPI at high concentrations showed direct bactericidal activity against L forms of Gram-positive bacteria including *Staphylococcus aureus* and *Streptococcus pyogenes*. This antimicrobial effect suggests that these proteins exert a cytotoxic effect on the cytoplasmatic membrane of these pathogenic bacteria [48]. Murine recombinant BPI 21 promotes the association of *Streptococcus pneumoniae* with murine macrophages through the binding of BPI to this Gram-positive bacterium [49]. Antimicrobial activity against Gram-positive bacteria was also found in GL13K, a modified peptide derived from SPLUNC 2 protein that showed bactericidal activity against *Streptococcus gordonii* [50].

The anti-angiogenic activity of BPI has been demonstrated in chorioallantoic membrane (CAM), which develops in close proximity to the eggshell membranes [16]. The CAM regulates the mobilization of calcium from eggshell to the chick embryo during development to provide the metabolic needs such as skeletal growth and neuromuscular activities [51]. However, we have no evidence whether OCX-36 regulates normal blood vessel development during chick embryonic growth.

We investigated whether OCX-36 binds LPS and LTA, which are bacterial cell wall components of Gram-negative and Gram-positive bacteria, respectively, and are known as pathogen associated molecular patterns (PAMPs). Some examples of PAMPs include lipoproteins, peptidoglycan, lipoteichoic acid (LTA), lipopolysaccharide (LPS) and lipoarabinomannan (LAM) [52]. Interaction with PAMPS such as LPS and/or LTA is a characteristic feature of the family of proteins with BPI/LBP/PLUNC–like domains [53]. OCX-36 showed significant binding of *E. coli* O111:B4 LPS, suggesting that it participates in innate host defense against bacterial challenge similar to BPI or LBP. OCX-36 also binds to LTA from *S. aureus* ATCC 6538, suggesting that its inhibition of S. aureus growth is likely dependent on its interaction with bacterial cell wall LTA.The ability to bind to LPS is also found in human PLUNC isoforms with that are present in nasal lavage fluid. This study identified SPLUNC 1 modified by N-linked glycosylation with LPS binding activity [54]. Parotid secretory protein (PSP), a PLUNC protein found in saliva is an LPS binding protein, as is a corresponding synthetic peptide (GL13NH2). The synthetic peptide showed anti-inflammatory property by inhibiting LPS stimulated secretion of tumor necrosis factor from macrophages [50].

In addition to its LPS binding activity, LBP also interacts with other PAMPs such as peptidoglycan and LTA. LBP binds to peptidoglycan breakdown products and lipopeptides and mediates innate immune responses [52]. In a murine meningitis experimental model, LBP was able to recognize peptidoglycan breakdown products derived from S. pneumoniae and modulate the inflammatory response [55]. LBP has been reported to modulate the effect of LTA. LBP might be has a dual role in the innate immune response since this protein is able to increase and decrease the effect of LTA in macrophages and monocytes [56]. All these studies report functional activities detected with recombinant purified LBP/BPI/PLUNC proteins.

### 3. OCX-36 SNP characterization and functions

Genetic variations in genes such as single nucleotide polymorphisms (SNPs) are associated with host susceptibility and resistance to infectious diseases. SNPs can have an impact on gene expression as well as the biological function of protein, which leads to phenotypic consequences [57]. SNPs in the LPB gene are associated with susceptibility to sepsis and multiple organ dysfunction [58], [59]. For example, a polymorphism in the human LBP gene affects the risk for Gram-negative bacteremia [60]. A polymorphism in the BPI gene (Lys/Glu - 216) was reported to be associated with increased risk for development of sepsis but is uncorrelated with gender; while a corresponding genetic variation in LBP is related to male gender [61]. A recent study showed that BPI gene polymorphism is associated with susceptibility to bowel disease [62]. An SNP in the SPLUNC1 gene that is associated with enhanced risk of nasopharyngeal carcinoma may be related to altered expression and binding affinity for specificity protein1 (Sp1) transcription factor [63].

OCX-36 SNPs were identified in the OCX-36 gene and one non-synomymous SNP was verified, coding for alternative amino acids at position 71 - Pro/Ser. To address the functional consequences of these alternative versions, OCX-36 was extracted and purified from eggshell membranes harvested from eggs laid by hens that were homozygous for either of the two OCX-36 alleles. The antimicrobial activity of OCX-36 polymorphic forms (Pro-71 and Ser-71) was assessed only against S. aureus ATCC 6538 due to limitations in the amount of purified OCX-36 available. Both OCX-36 versions were inhibitory against growth of S. aureus ATCC 6538; no significant difference between them was found. Purified OCX-36 (proline-71) and (serine-71) bind to E. coli O111:B4 LPS and to LTA from *S. aureus* ATCC 6538. The Pro-71 form binds significantly more strongly to LTA than the Ser-71 form; purified OCX-36 with a mixture of both forms was intermediate. The mechanism by which these alterations in amino acid at position 71 affect the LTA binding activity of OCX-36 is likely due to differences in tertiary structure due to the significant differences between the properties of serine and proline.

### 4. Proposed model for OCX-36 function

OCX-36 is specifically expressed in the chicken reproductive and digestive tracts. Our proposal for OCX-36 function is that it is a pattern recognition molecule, which recognizes bacterial endotoxins as a first step to eliminate pathogens. However, a future study to determine if OCX-36 competes for LPS binding or the interaction of OCX-36 with Toll-like receptors (TLR-2 and TLR-4) is necessary for a better understanding of the mechanism of OCX-36 action regarding our hypothetical model for OCX-36 as a pattern recognition receptor.
